# Developing Robust Human Liver Microsomal Stability Prediction Models: Leveraging Inter-Species Correlation with Rat Data

**DOI:** 10.3390/pharmaceutics16101257

**Published:** 2024-09-27

**Authors:** Pranav Shah, Vishal B. Siramshetty, Ewy Mathé, Xin Xu

**Affiliations:** National Center for Advancing Translational Sciences (NCATS), 9808 Medical Center Drive, Rockville, MD 20850, USA

**Keywords:** metabolic stability, human liver microsomes, rat liver microsomes, quantitative structure activity relationships, in silico ADME

## Abstract

**Objectives:** Pharmacokinetic issues were the leading cause of drug attrition, accounting for approximately 40% of all cases before the turn of the century. To this end, several high-throughput in vitro assays like microsomal stability have been developed to evaluate the pharmacokinetic profiles of compounds in the early stages of drug discovery. At NCATS, a single-point rat liver microsomal (RLM) stability assay is used as a Tier I assay, while human liver microsomal (HLM) stability is employed as a Tier II assay. We experimentally screened and collected data on over 30,000 compounds for RLM stability and over 7000 compounds for HLM stability. Although HLM stability screening provides valuable insights, the increasing number of hits generated, along with the time- and resource-intensive nature of the assay, highlights the need for alternative strategies. One promising approach is leveraging in silico models trained on these experimental datasets. **Methods:** We describe the development of an HLM stability prediction model using our in-house HLM stability dataset. **Results:** Employing both classical machine learning methods and advanced techniques, such as neural networks, we achieved model accuracies exceeding 80%. Moreover, we validated our model using external test sets and found that our models are comparable to some of the best models in literature. Additionally, the strong correlation observed between our RLM and HLM data was further reinforced by the fact that our HLM model performance improved when using RLM stability predictions as an input descriptor. **Conclusions:** The best model along with a subset of our dataset (PubChem AID: 1963597) has been made publicly accessible on the ADME@NCATS website for the benefit of the greater drug discovery community. To the best of our knowledge, it is the largest open-source model of its kind and the first to leverage cross-species data.

## 1. Introduction

Before the turn of the century, pharmacokinetic problems were one of the primary reasons for drug attrition, responsible for around 40% of all failures [1]. To address this, various high-throughput in vitro assays, such as metabolic stability, have been developed to assess pharmacokinetic properties of compounds during early stages of drug discovery [2,3]. Hepatic metabolic stability is crucial in drug discovery, affecting both oral bioavailability and compound elimination. The cytochrome P450 (CYP450) enzyme family plays a major role in xenobiotic metabolism. Among these, CYP3A4 is the most significant, metabolizing around 50% of known xenobiotics in humans [4,5]. Typically, an in vitro stability assay using HLM is the standard method for estimating clearance in humans. The results from this assay are utilized to rank and prioritize compounds for further development in the drug discovery pipeline. 

We, at the National Center for Advancing Translational Sciences (NCATS), employ a single-point RLM stability assay as a Tier I screening tool, with RLM stability data being generated for every compound synthesized at NCATS. For Tier II, a high-throughput multi-point HLM stability assay is utilized. Over the last few years, we have screened >30,000 compounds from more than 300 projects for RLM stability and >7000 compounds from more than 100 discovery and development projects have been screened for HLM stability. One of the goals of this study is to compare how well our Tier I RLM data correlate with Tier II HLM data. This correlation will help us understand whether our screening paradigm needs to be altered.

Drug discovery is a long, expensive venture, and costs continue to escalate with time [6,7]. Thus, any innovations that cut time or reduce costs are highly valuable. In silico machine learning approaches have gained popularity as well as success and predictive models are routinely employed in major pharmaceutical companies. Results from these predictive models are used as a rank-ordering mechanism for prioritizing compound synthesis. While open-access HLM stability models exist, they are usually developed using small datasets or with data sourced from the literature, which can induce error due to variability in experimental protocols, microsomal vendor differences, and drug/enzyme concentration differences. Commercially available HLM models are expensive and not exempt from the above-mentioned disadvantages. In this study, we employed traditional and advanced machine learning techniques to develop quantitative structure activity relationship (QSAR) models for predicting HLM stability. Additionally, we identified a strong correlation between our RLM and HLM data. This correlation was further reinforced by the observation that the accuracies and predictive performance of our HLM model improved when RLM stability predictions were included as an input descriptor. This improvement underscores the robustness and reliability of the relationship between these two datasets. We also compared our models with other equivalent models in literature and found that our models achieved comparable balanced accuracies. Moreover, the best model from our study, which boasts an accuracy of 80%, has been made publicly accessible on the NCATS ADME portal (https://opendata.ncats.nih.gov/adme/, accessed on 31 July 2024). The in silico model derived from this dataset will be a valuable resource for accelerating translational research across diverse drug discovery institutions.

## 2. Materials and Methods

### 2.1. Microsomal Stability Assay

The substrate depletion method was used to determine half-life (t_1/2_) of compounds by measuring the disappearance of the parent compound over time. Incubations were performed on a Tecan EVO 200 robotic system (Morrisville, NC, USA), equipped with a 96-channel head, Inheco heating block, and controlled by EVOware software (Version 3.5). Mixed-gender human liver microsomes were purchased from Xenotech (Kansas City, KS, USA) (Catalog: H0610). Gentest NADPH Regenerating Solution A and B (Catalog: 451220/451200), Axygen reservoirs (Catalog: RES-SW384-LP/RES-SW384-HP) were purchased from Corning Inc. (Corning, NY, USA). Incubation plates (384-well, 250 µL; Catalog# 186002632) and LC/MS analysis plates (384-well, 100 µL; Catalog# 186002631) were purchased from Waters Inc. (Milford, MA, USA). The compounds used for assay controls, internal standards, and buffers including albendazole, buspirone, propranolol, loperamide, antipyrine, potassium phosphate monobasic, and potassium phosphate dibasic were purchased from Sigma-Aldrich (St. Louis, MO, USA). An albendazole solution in acetonitrile (ACN/IS) was prepared for use as an internal standard. Each 110 μL reaction mixture included the test compound (1 μM), HLM (0.5 mg/mL), and NADPH regenerating system in phosphate buffer (pH 7.4). The samples were incubated in 384-well plates at 37 °C for 0, 5, 10, 15, 30, and 60 min. At each designated time point, 10 μL of the mixture was transferred to another 384-well plate containing cold ACN/IS. The plates were then centrifuged at 3000 rpm for 20 min at 4 °C, and supernatants were collected into a 384-well injection plate. Sample quantification was performed using Thermo UPLC/HRMS and data were analyzed using TraceFinder software (Version 4.1). The data were then extracted, and half-life analysis was performed using our in-house Validator software (Version 1.0) as described previously [8,9].

### 2.2. HLM Stability Dataset

The raw dataset was preprocessed to generate training and test data for the purpose of building and validating prediction models. Compounds were classified as unstable (t_1/2_ < 30 min) or stable (t_1/2_ > 30 min). Compound structures were normalized [10] and LyChI identifiers (https://github.com/ncats/lychi, accessed on 31 July 2024) were generated for all standardized structures to identify unique compounds. After omitting duplicates and compounds with contrasting experimental results, the final processed dataset comprised a total of 6648 (unstable: 2197; stable: 4451) compounds. 

### 2.3. Modeling Methods

#### 2.3.1. Random Forest

Random forest is an ensemble learning method primarily used for classification and regression tasks. It operates by constructing a multitude of decision trees during training and outputting the mode of the classes (classification) or mean prediction (regression) of the individual trees. Each tree in a random forest is built from a random subset of the training data through a technique known as bootstrap aggregation, or bagging. Additionally, during the construction of each tree, a random subset of features is selected at each split point, which helps in reducing the variance and avoiding overfitting. The overall performance of random forest is robust due to its ability to generalize well to unseen data, making it particularly useful in high-dimensional spaces often encountered in drug discovery. The method was introduced by Breiman in 2001 and has since become a cornerstone in machine learning applications due to its simplicity and effectiveness [11].

#### 2.3.2. XGBoost

XGBoost, short for eXtreme gradient boosting, is a scalable and efficient implementation of gradient boosting machines, which are ensemble learning methods that create a model in a stage-wise fashion from weak learners, typically decision trees. It was developed by Chen and Guestrin in 2016 to address the need for a more powerful and computationally efficient gradient boosting framework [12]. XGBoost utilizes second-order gradient information, advanced regularization (L1 and L2), and a distributed computing paradigm to build robust predictive models that can handle sparse data and large-scale datasets, common in drug discovery research. Its implementation includes various optimizations such as tree pruning, parallel processing, and cache awareness, which collectively enhance its speed and performance. The algorithm’s predictive power and flexibility have made it a favorite in many data science competitions and practical applications.

#### 2.3.3. Graph Convolutional Neural Network (GCNN)

Graph Convolutional Neural Networks (GCNNs) are a class of neural networks designed to operate directly on graph-structured data, making them particularly well suited for tasks involving small molecules, which can be naturally represented as graphs where atoms are nodes and bonds are edges. The key idea behind GCNNs is to perform convolution operations on graphs by aggregating information from a node’s neighbors, enabling the network to learn hierarchical feature representations. This is especially advantageous in drug discovery, as the network can effectively capture the spatial and chemical properties of molecules, leading to accurate predictions of molecular properties such as solubility, toxicity, and binding affinity.

GCNNs leverage message passing, where each node iteratively updates its representation by aggregating and transforming information from its neighbors. These updates are typically parameterized by learnable weight matrices, allowing the network to learn relevant patterns and relationships within the molecular graph. Kipf and Welling laid the foundation for modern GCNN architectures, demonstrating their potential in various applications involving graph data [13]. The Chemprop Python library is a widely used tool in the computational chemistry and drug discovery communities for implementing GCNNs. It provides a user-friendly interface for training GCNN models on molecular data, facilitating the prediction of chemical properties and bioactivities. Chemprop builds on the foundational principles of GCNNs and offers optimized implementations that cater to the specific needs of small-molecule analysis [14].

### 2.4. Model Building and Validation

Cross-validation using a random split is a common technique for assessing the generalizability of a machine learning model. In this method, the training data are randomly divided into internal training and internal validation subsets multiple times. For each split, the model is trained on the internal training subset and evaluated on the internal validation subset. This process helps ensure that the model’s performance is not overly dependent on a particular partition of the data. The random split method is particularly useful in drug discovery applications where datasets can be limited in size and diverse in nature. By averaging the performance metrics across multiple splits, one can obtain a more reliable estimate of the model’s ability to generalize to unseen data. This technique helps in mitigating overfitting and provides a robust measure of the model’s predictive power. In this study, we performed a 5-fold cross-validation (5-CV). This step is followed by external validation.

External validation involves testing the trained model on an entirely independent dataset that was not used during the training or internal validation phases. This step is crucial in drug discovery to ensure that the model’s predictions are genuinely generalizable and not just a result of overfitting to the training data. External validation provides a stringent test of the model’s performance, as it simulates real-world application scenarios where new, unseen molecules are encountered. The best models from internal validation were validated on the NCATS’s external validation set and the three external datasets. The following metrics were used in order to measure and compare the performance of the models and are based on the four elements of a confusion matrix: true positives (TN), i.e., those positive class compounds correctly predicted as positive; false positives (FP), i.e., those negative class compounds incorrectly predicted as positive; true negatives (TN), i.e., those negative class compounds correctly predicted as negative; false negatives (FN), i.e., those positive class compounds incorrectly predicted as negative. 

Accuracy: Accuracy is the ratio of correctly predicted instances to the total instances in the dataset [15]. It provides a straightforward measure of the model’s overall performance, but it can be misleading in imbalanced datasets where one class dominates.

Accuracy = (TP + TN)/(TP + FP + TN + FN)

Sensitivity (or Recall): Sensitivity, also known as recall or true positive rate, measures the proportion of actual positives that are correctly identified by the model. It is critical in contexts where missing positive cases is particularly costly.

Sensitivity = TP/(TP + FN)

Specificity: Specificity, or true negative rate, measures the proportion of actual negatives that are correctly identified by the model. It is important in contexts where false positives are costly.

Specificity = TN/(TN + FP)

Positive Predictive Value (PPV): PPV, or precision, measures the proportion of positive predictions that are actually correct. It indicates the reliability of positive predictions made by the model.

PPV = TP/(TP + FP)

Negative Predictive Value (NPV): NPV measures the proportion of negative predictions that are actually correct. It indicates the reliability of negative predictions made by the model.

NPV = TN/(TN + FN)

Area Under the Receiver Operating Characteristic Curve (AUC-ROC): The AUC-ROC measures the model’s ability to distinguish between positive and negative classes across various threshold settings [16]. It plots the true positive rate (sensitivity) against the false positive rate (1-specificity) and calculates the area under this curve. A higher AUC indicates better model performance.

Cohen’s Kappa: Cohen’s Kappa [17] is a statistical measure that compares an observed accuracy with an expected accuracy (random chance). It accounts for the possibility of agreement occurring by chance, providing a more robust metric for evaluating classification models, especially with imbalanced datasets.

TP, TN, FP, and FN are the numbers of true positive predictions, true negative predictions, false positive predictions, and false negative predictions, respectively.

## 3. Results and Discussion

### 3.1. Assay Performance

Four control compounds were run with each plate. The assay reproducibility data for these control compounds over 6 years spanning > 40 plates are shown in Table 1. Since the t_1/2_ data were capped at 120 min, standard deviation for the high stability control, i.e., antipyrine was not calculated.

### 3.2. Distribution of HLM Data

**Figure 1:**
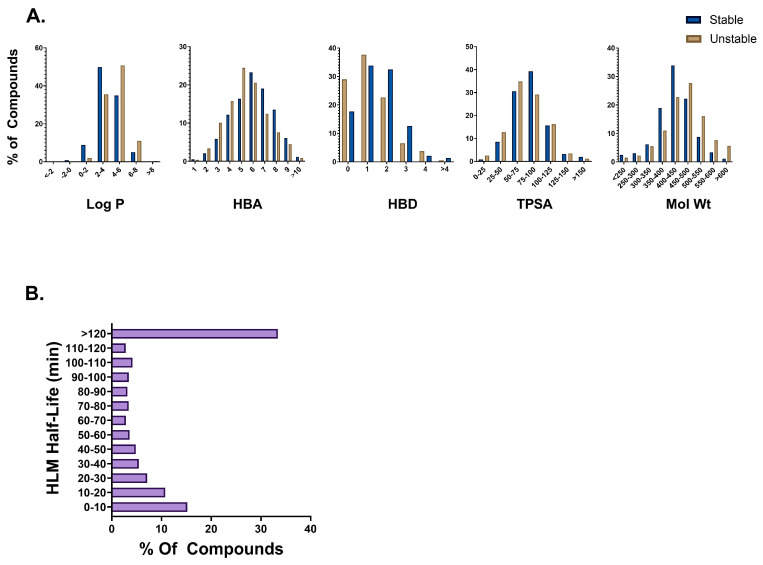
(**A**) Molecular property distribution plots for stable (half-life > 30) and unstable (half-life < 30 min) compounds in the HLM dataset. (**B**) Half-life distribution of compounds in the HLM dataset.

The final 6648 compound HLM dataset was heavily skewed towards the stable class (67%) compared to the unstable class (33%) (Figure 1B). The largest number of compounds (33%) fell into the >120 min bin. To understand the diversity of our dataset, distributions based on t_1/2_ and molecular properties including log P, total polar surface area (TPSA), molecular weight, hydrogen bond acceptor (HBA), and donors (HBD) were scrutinized (Figure 1A). The majority of the compounds in our dataset belong in the 350–500 molecular weight range; have log P values between 2 and 6; TPSA between 50 and 125; and have between 3 and 8 HBAs and 0 and 2 HBDs. These molecular property distributions indicate wide diversity in our dataset. Interestingly, we see that unstable compounds tended to have slightly lower HBA and TPSA values whereas unstable compounds tended to have slightly higher log P values and molecular weights. No differences in HBD distributions were found between the two classes. A statistical analysis revealed significant differences in these properties between the stable and unstable compounds (Table S1).

### 3.3. Microsomal Stability Screening Paradigm at NCATS

**Figure 2:**
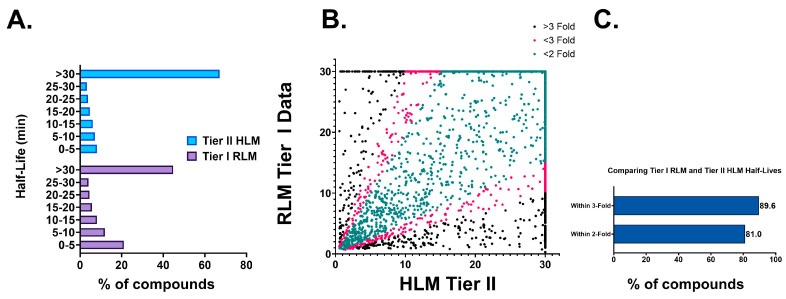
(**A**) Half-life distribution of overall Tier I RLM and Tier II HLM datasets. (**B**) Comparison of fold differences in half-lives between overlapping compounds in Tier I RLM and Tier II HLM datasets. (**C**) Percentage of compounds with less than 2-fold and 3-fold half-life variability between Tier I RLM and Tier II HLM datasets.

Every compound synthesized at the NCATS undergoes a Tier I single time point (15 min) RLM stability assessment [18]. Typically, after optimizing Tier I RLM stability, compounds move into Tier II stability testing where they are screened in a multi-time point (0–60 min) assay in rat, human, and other species. This pattern is clearly demonstrated by the half-life distribution data in Figure 2A. A total of 55% of compounds were unstable in the Tier I RLM assay (total dataset > 35,000), whereas the corresponding number for the Tier II HLM assay (total dataset 6648) was 33%. 

Since our Tier I RLM and Tier II HLM assays have different extrapolated maximum half-lives, 30 min and 120 min, respectively, Tier II HLM half-lives were capped at 30 for correlation analysis. Despite variations in the assay and species, our analysis reveals that 81% of the compounds exhibited less than 2-fold differences, and approximately 90% of the compounds showed less than 3-fold differences (Figure 2B,C) in half-life values between the RLM and HLM fractions. This validates our rationale for selecting RLM as the matrix for Tier I screening.

### 3.4. Five-Fold Cross-Validation

In this study, we employed 5-fold cross-validation (5-CV) to rigorously evaluate the performance of RF, XGBoost, and GCNN methods. RDKit descriptors were used as the default features for the RF and XGBoost methods, providing a baseline for comparison. In contrast, the GCNN method utilized a combination of RDKit descriptors and graph featurization, integrating additional features to capture more intricate molecular relationships. While all three methods showed comparable performance with the RDKit descriptors, XGBoost tended to provide a better balance between sensitivity and specificity (Table 2). GCNN, in comparison to the two baseline methods, demonstrated on average higher specificity and poor sensitivity. The sensitivity improved slightly when RDKit features were used as additional features. Overall, the baseline XGBoost method provided the best cross-validation performance (across different metrics) when using RDKit descriptors as features. For each method, we also employed a grid search method to assess a number of hyperparameter combinations. In the case of the GCNN method, the inbuilt Bayesian optimization was employed instead of the grid search method. The parameters of the XGBoost model that offered the best performance are listed in Table S2 of the Supplementary Information.

In addition to employing RDKit descriptors, we incorporated predictions from our previously published in silico RLM stability prediction model as an additional descriptor. This approach is grounded in the rationale that RLM stability correlated strongly with HLM stability as described above. While the use of biological outcomes as descriptors in QSAR models is well established [19,20], this is the first study that reports leveraging RLM stability model predictions as descriptors for the development of an HLM model. This novel integration was expected to add a unique layer of biological relevance and potentially enhance the accuracy of HLM stability predictions by identifying more true positives (i.e., unstable compounds). As anticipated, adding RLM stability predictions boosted the sensitivity of the models from all three methods, without impacting their specificity, leading to an improvement in overall accuracy. Again, XGBoost remains the best-performing method with an average sensitivity of 70% and specificity of 89%.

### 3.5. External Datasets

While there are several HLM QSAR publications, most of them have been developed either using data from literature or using proprietary data where neither the external test set nor the models are publicly available. However, we were able to find three studies/datasets that are similar to the scope of our study and could be used for comparison (Table 3). The first study came out of Genentech where they used a 20,000-compound proprietary dataset to develop an HLM QSAR model and used 972 compounds from ChEMBL as an external test set (E1) to validate their model performance. The external test set was compiled from 25 individual ChEMBL datasets where the compound concentration varied from 0.5–1 µM and the microsomal protein concentration varied from 0.25–1 mg/mL [21]. The second study was conducted by AstraZeneca, where they employed their in-house experimental method to test 1102 known compounds (E2). The data from this study was then made available for developing predictive models or for benchmarking purposes. The data are publicly available on the ChEMBL website (CHEMBL3301370) and assay details were obtained from Sternbeck et al., 2010 [22]. The third study was by Ryu et al., 2022 where they developed a model using ~2000 proprietary compounds and used a 61-compound external test set (E3) for model validation [23]. We used the E1 and E3 external test sets to compare model performance metrics whereas the E2 dataset was simply used as an external test set to validate our model. 

**Figure 3:**
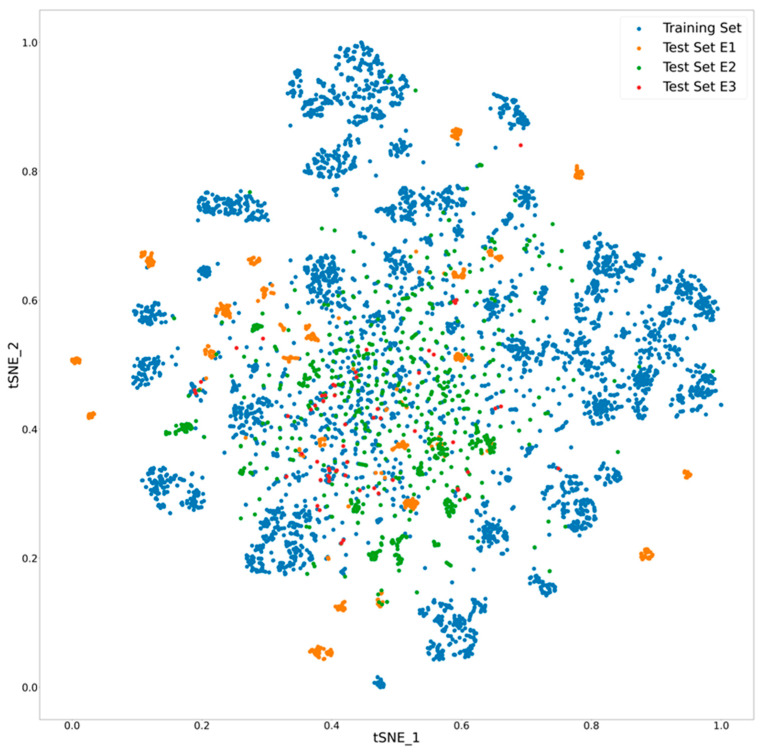
Chemical space visualization of external test sets in comparison to the internal training set. The *x* and *y* axes indicate the first two dimensions (tSNE_1 and tSNE_2) of the t-SNE embedding.

To compare the chemical space coverage between the three external test sets and our dataset, the compounds were projected into a low-dimensional space using the t-distributed Stochastic Neighbor Embedding (t-SNE) method (Figure 3) as described previously [18]. Since the compounds in these external test sets were obtained from literature, there is significant overlap in the chemical space; however, distinct clusters of compounds are seen in the Genentech and the AZ datasets. Although the external test sets appear diverse, the NCATS dataset seems to have reasonable diversity and a good chance of accurately predicting stability of these compounds.

### 3.6. External Validation

Based on our cross-validation results, we selected the top-performing method, i.e., XGBoost, to assess its performance across three external test sets: E1, E2, and E3. GCNN was also chosen for this analysis due to its consistent performance in our previously published ADME models [6,18,24]. Since both RF and XGBoost are tree-based methods, we decided not to continue evaluating the RF method. For test sets E1 and E3, predictions from the best model were provided in the original studies. Consequently, we compared the results of our models with the performance of the best model from those respective studies.

Before this, given the strong correlation between our Tier I RLM and Tier II HLM data, we assessed the performance of our previously published RLM stability prediction model in forecasting the HLM stability of compounds in the external datasets. (Table 4). The RLM model was able to identify 77% and 86% of unstable compounds in datasets E1 and E2, respectively. While the RLM model was able to correctly identify all unstable compounds in the E3 dataset, the majority of compounds from this dataset were classified as unstable, meaning the RLM predictions resulted in a poor specificity. A high number of RLM predictions classifying the external dataset compounds as unstable could be attributed to the dominance of unstable compounds in our Tier I RLM dataset. The RLM stability training dataset predominantly comprised early-stage compounds and a lot of them become deprioritized due to poor metabolic stability. Therefore, we believe that using RLM predictions as an additional descriptor could have a positive impact on the sensitivity of the models without impacting the specificity.

The HLM XGBoost model based on RDKit descriptors combined with RLM predictions consistently stood out as the best-performing model on all three external datasets. The Supplementary Information Figure S1 lists the top 20 RDKit descriptors selected by the XGBoost model. As anticipated, adding RLM predictions contributed to improved sensitivity of the HLM models (Table 5). Particularly, the XGBoost model benefited from the RLM predictions more than the GCNN model. The difference in performance between XGBoost and GCNN, particularly in how they leverage the biological descriptor (RLM stability prediction), can be attributed to the nature of the models and how they handle features. XGBoost can directly utilize the biological descriptor as an individual feature, and since decision trees can handle various types of numerical data well, this descriptor can significantly enhance model performance if it is highly correlated with the target. Also, XGBoost offers a natural way to measure feature importance, allowing it to effectively weigh the biological descriptor more if it is found to be highly predictive. On the other hand, GCNNs primarily excel at learning from the structure and connections within the graph data. The neural network’s architecture is inherently more focused on extracting patterns from graph-structured information rather than individual numerical descriptors. While GCNNs can incorporate additional numerical descriptors, these features may not be utilized as effectively as in tree-based models. The integration of such features into the graph-based learning process might dilute the influence of the biological descriptor compared to how XGBoost can directly prioritize it. Moreover, training GCNNs involves complex interactions between the graph structure and the additional numerical features. Since the biological descriptor is not seamlessly integrated into the graph’s representation, its predictive power might be underutilized. While adding RLM stability predictions as a descriptor impacted the specificity of the GCNN model across all external datasets, the XGBoost model demonstrated an improvement in both sensitivity and specificity on the E2 and E3 datasets. The ROC curves for the best-performing XGBoost and GCNN models are provided in the Supplementary Information (Figure S2).

The best NCATS model, i.e., XGBOOST model with RDKit descriptors and RLM predictions, performed as well as the Genentech model on the E1 external test set despite being a third in size. It also performed better than the PredMS model on the E3 external test set. Thus, we deployed this model on the ADME@NCATS website. While a lot of commercial HLM stability models exist, there is a dearth of open-source HLM stability prediction models (Table 6). Several other websites and models exist, but they all utilize data from ChEMBL. We have used FP-ADMET to represent these platforms. To the best of our knowledge, our model is the largest open-source model developed using data generated in the same lab, following the same method, eliminating protocol and lab-to-lab variabilities. There have been a handful of studies that have developed multi-species microsome stability models; however, they have either attempted to develop species-specific models [25,26] or developed consensus models based on the individual species models to improve performance [27]. As far as we are aware, this is the first study of its kind to leverage cross-species data as a descriptor to improve the performance metrics of a microsome stability model.

Although our model avoids the common drawbacks associated with HLM models built from compiled literature data, there are still important factors to consider when interpreting its predictions. QSAR models typically rely on simplified descriptors and do not consider key factors that impact metabolic stability like the interaction of the test compound with the enzyme, allosteric effects, or chirality. Additionally, even though our dataset is diverse, predictions for compounds that are structurally different from our training dataset could be less reliable. As NCATS regularly undertakes new projects, our chemical space will continually grow and expand. We are currently working on a backend framework for our in silico website that would allow us to continuously update our models with newly generated data. Since majority of compounds from our training set are part of currently ongoing projects, we are unable to make the entire dataset public; however, a 900-compound subset of the data has been deposited on PubChem (AID: 1963597). As projects wind down and patents are cleared, we plan to deposit additional data on PubChem, expanding the public dataset.

## 4. Conclusions

In this study, we describe the development, validation, and dissemination of an in silico HLM stability model. This model is useful for early-stage drug discovery, allowing researchers to identify compounds with favorable metabolic profiles and avoid those that may be rapidly degraded in the liver. Furthermore, we observed a significant correlation between our RLM data and our HLM data. By incorporating the stability predictions from our RLM data, the accuracy and predictive capabilities of our HLM model were notably improved, underscoring the robustness of the relationship between these two datasets. To the best of our knowledge, it is the largest open-source model developed using data generated from a single laboratory using a single protocol as well as the first model of its kind that leverages cross-species data. The best-performing model, i.e., XGBoost with RDKit descriptors and RLM predictions, along with a subset of our dataset, has been hosted on the NCATS in silico ADME website (https://opendata.ncats.nih.gov/adme/, accessed on 31 July 2024) to benefit the drug discovery community. 

## Figures and Tables

**Table 1:** Assay reproducibility for control compounds—mean half-life and S.D values are reported. Since data are not extrapolated beyond 120 min, S.D was not calculated for antipyrine.

Compound	Half-Life (t_1/2_) in Minutes
Buspirone	14.4 ± 4.1
Loperamide	18.9 ± 5.2
Propranolol	55.2 ± 10.1
Antipyrine	>120

**Table 2:** Five-Fold cross-validation performance of the three methods based on different descriptors.

Method	Descriptor(s)	AUC	Accuracy	Sensitivity	Specificity
RF	RDKit	0.87 ± 0.02	0.81 ± 0.02	0.55 ± 0.04	0.93 ± 0.02
RF	RDKit + RLM	0.89 ± 0.01	0.83 ± 0.01	0.62 ± 0.04	0.93 ± 0.01
XGBoost	RDKit	0.87 ± 0.02	0.81 ± 0.02	0.65 ± 0.02	0.89 ± 0.02
XGBoost	RDKit + RLM	0.89 ± 0.02	0.83 ± 0.01	0.70 ± 0.03	0.89 ± 0.02
GCNN	Graph	0.84 ± 0.02	0.80 ± 0.02	0.57 ± 0.07	0.91 ± 0.03
GCNN	Graph + RDKit	0.86 ± 0.02	0.80 ± 0.02	0.62 ± 0.07	0.89 ± 0.03
GCNN	Graph + RDKit + RLM	0.87 ± 0.03	0.82 ± 0.02	0.67 ± 0.05	0.89 ± 0.02

**Table 3:** Details of external test sets along with their sources.

External Test Set	Total Molecules	Unstable	Stable
E1 (Genentech)	972	544	428
E2 (AstraZeneca)	1102	260	842
E3 (PredMS)	61	12	49

**Table 4:** Performance of RLM stability prediction model on the three external datasets. N/A: not applicable.

Dataset	AUC	Accuracy	Sensitivity	Specificity
E1 (NCATS Results)	0.69	0.66	0.77	0.52
E1 (Genentech Results)	N/A	0.67	0.67	0.68
E2 (NCATS Results)	0.62	0.78	0.86	0.54
E3 (NCATS Results)	0.54	0.80	1.00	0.43
E3 (PredMS Results)	N/A	0.74	0.70	0.86

**Table 5:** Performance of NCATS XGBoost-HLM and GCNN-HLM models with and without using RLM predictions as a descriptor on the three external test sets. N/A: not applicable.

Test Set	Model (Descriptors)	Accuracy	AUC	Sensitivity	Specificity
E1	NCATS XGBoost (RDKit)	0.64	0.69	0.58	0.72
E1	NCATS XGBoost (RDKit + RLM)	0.66	0.73	0.66	0.66
E1	NCATS GCNN (RDKit)	00.67	0.70	0.62	0.73
E1	NCATS GCNN (RDKit + RLM)	0.67	0.77	0.65	0.70
E1	Genentech Model	0.67	N/A	0.67	0.68
E2	NCATS XGBoost (RDKit)	0.67	0.72	0.66	0.68
E2	NCATS XGBoost (RDKit + RLM)	0.73	0.80	0.75	0.73
E2	NCATS GCNN (RDKit)	0.74	0.77	0.62	0.78
E2	NCATS GCNN (RDKit + RLM)	0.76	0.68	0.62	0.70
E3	NCATS XGBoost (RDKit)	0.74	0.84	0.50	0.80
E3	NCATS XGBoost (RDKit + RLM)	0.84	0.87	0.75	0.86
E3	NCATS GCNN (RDKit)	0.82	0.87	0.50	0.90
E3	NCATS GCNN (RDKit + RLM)	0.85	0.79	0.58	0.84
E3	PredMS Model	N/A	0.74	0.70	0.86

**Table 6:** Open-source HLM stability models in the literature.

Website	Number of Compounds Used to Train Model	Source of Data	Model Availability	Data Availability	Accuracy of Training Sets
PredMS	1917	Own	Yes/Website	External Set/61 compounds	ACC: 0.68
FP-ADMET	3654	ChEMBL	Yes/Downloadable Offline version on Github	Yes/ChEMBL	BACC: 0.77
ADME@NCATS	6648	Own	Yes/Website and Downloadable Offline version on Github	Yes/Partial dataset on PubChem (AID: 1963597)	BACC: 0.80

## Data Availability

The model is available on the ADME@NCATS website and a subset of the data has been deposited into PubChem (AID: 1963597).

## Supplementary Materials

The following supporting information can be downloaded at: https://www.mdpi.com/article/10.3390/pharmaceutics16101257/s1, Figure S1: Top 20 important features learned by the best XGBoost model; Figure S2: ROC curves for the best performing XGBoost and GCNN models on the three external datasets, E1, E2 and E3; Table S1: Statistical analysis of differences in physicochemical properties between stable and unstable compounds; Table S2: Hyperparameter search space and the best performing parameters of the XGBoost model.
