# Supplementary material for: Developing Robust Human Liver Microsomal Stability Prediction Models: Leveraging Inter-Species Correlation with Rat Data
Source: Pharmaceutics. 2024 Sep 27;16(10):1257. doi: 10.3390/pharmaceutics16101257 (PMC11510424; doi:10.3390/pharmaceutics16101257)
Supplement: Supplementary file 1 [file pharmaceutics-16-01257-s001.zip › pharmaceutics-3163536-supplementary.pdf]

Supplementary Information

Developing Robust Human Liver Microsomal Stability Prediction Models: Leveraging Inter-Species Correlation with Rat Data

Pranav Shah *, Vishal B. Siramshetty, Ewy Mathé and Xin Xu

Figure S1. Top 20 important features learned by the best XGBoost model.

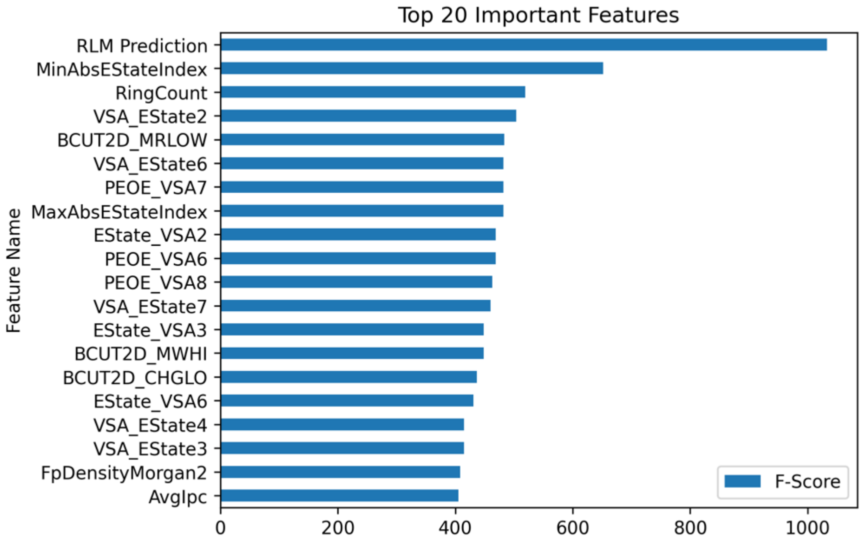

Figure S2. ROC curves for the best performing XGBoost and GCNN models on the three external datasets, E1, E2 and E3.

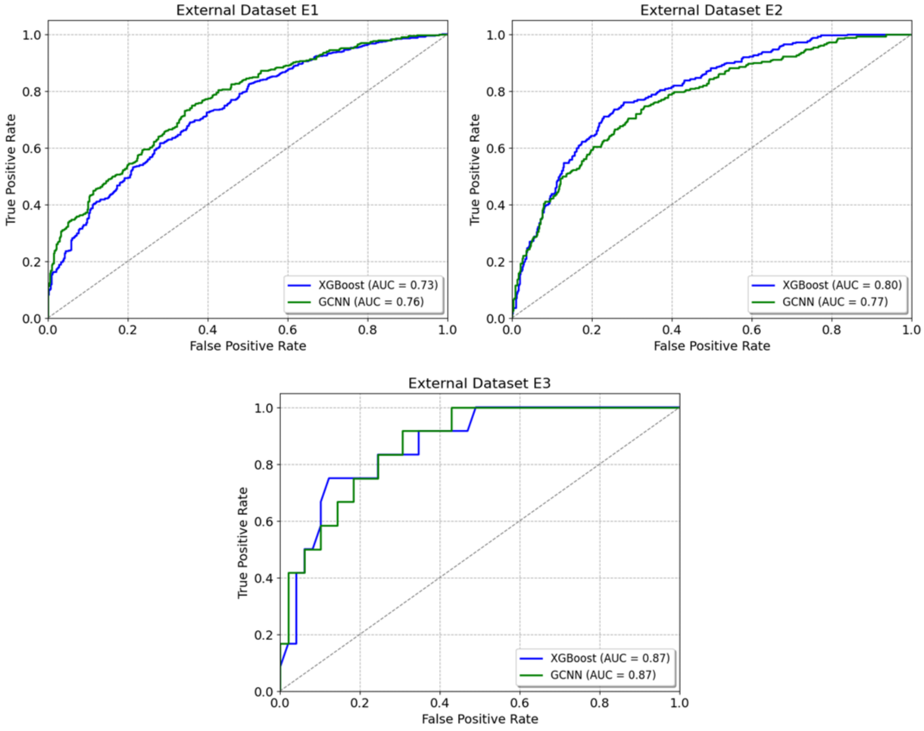

Table S1. Statistical analysis of differences in physicochemical properties between stable and unstable compounds.

Property	T-Test p-value	Mann-Whitney U-Test p-value
**Log P**	5.68E-96	2.26E-91
**HBA**	1.43E-34	3.65E-39
**HBD**	3.98E-29	7.80E-36
**TPSA**	2.57E-07	3.06E-07
**Mol Wt**	2.40E-56	5.50E-64

Table S2. Hyperparameter search space and the best performing parameters of the XGBoost model.

Parameter Name	Search Space (best performing parameter in bold)
**n_estimators**	100, **300**, 500
**max_depth**	2, 4, 6, 8, **10**
**learning_rate**	0.01, **0.05**, 0.1, 0.2, 0.3
**subsample**	0.1, 0.2, **0.3**, 0.4, 0.5, 0.6, 0.7, 0.8, 0.9, 1.0
